# Fat body, fat pad and adipose tissues in invertebrates and vertebrates: the nexus

**DOI:** 10.1186/1476-511X-13-71

**Published:** 2014-04-23

**Authors:** Odunayo Ibraheem Azeez, Roy Meintjes, Joseph Panashe Chamunorwa

**Affiliations:** 1 Department of Anatomy and Physiology, Faculty of Veterinary Science, University of Pretoria, Private Bag X04, Onderstepoort 0110 Pretoria, South Africa; 2 Department of Veterinary Physiology, Biochemistry and Pharmacology, Faculty of Veterinary Medicine, University of Ibadan, Ibadan, Nigeria

**Keywords:** Fat body, Adipose tissue, and Metabolic syndrome

## Abstract

The fat body in invertebrates was shown to participate in energy storage and homeostasis, apart from its other roles in immune mediation and protein synthesis to mention a few. Thus, sharing similar characteristics with the liver and adipose tissues in vertebrates. However, vertebrate adipose tissue or fat has been incriminated in the pathophysiology of metabolic disorders due to its role in production of pro-inflammatory cytokines. This has not been reported in the insect fat body. The link between the fat body and adipose tissue was examined in this review with the aim of determining the principal factors responsible for resistance to inflammation in the insect fat body. This could be the missing link in the prevention of metabolic disorders in vertebrates, occasioned by obesity.

## Introduction

Living organisms, probably by virtue of limited resources for survival, are intuitionally wired with the ability to conserve available resources. This feature is not limited to any specific phyla, gender or any other status, but common along the phylogenetic tree. It is also amazing that the degree of sophistication in the process of conservation increases as the organism advances in the evolutionary tree.

The basic elements that are conserved are food, in the form of energy sources for metabolic and reproductive activities and water. Several experiments have shown that there is a limit to which an organism can survive whilst still maintaining normal physiological activities if food is deprived [1,2]. Though some extreme cases of survival without food have been documented, for example some species of penguins do not eat while incubating their eggs, which could be for several months. Similarly, Pacific salmon do not feed during their upstream migration [2]. Eels do not feed during their migration across the Atlantic, in preparation for spawning in fresh water habitat like the salmon, migrating many thousands of kilometers over a year without feeding and may survive lack of food for many years during estivation [3]. According to Boetius and Boetius, [4] and Olivereau and Olivereau [5], eels have been found to survive starvation of up to four years, surviving basically on oxidation of stored fats and even amino acid derived from body proteins for gluconeogenesis.

Nutrients are stored in the form of fat, (which are esters of fatty acid and glycerol) and glycogen (a branched polymer of α-D-glucose) in tissues for use during period of scarcity of these resources. Storage and use of fat is, however, a more efficient and economical method of energy conservation because β oxidation of fatty acid yields more energy in terms of number of ATP molecules as fatty acid is converted to carbon dioxide and water.

In the fed state or during the period of food availability and abundance, the major energy substrate is glucose, which is broken down to pyruvate and lactate in aerobic and anaerobic conditions, respectively. Excess amino acid is deaminated to form acetyl CoA and urea while fatty acid and glycerol from fat digestion as well as pyruvate and lactate are also converted to acetyl CoA. Acetyl CoA, could either be used to produce energy in the form of ATP via the citric acid cycle or converted to triacylglycerol for storage as fat by carboxylation in the presence of bicarbonate and biotin to form malonyl CoA (the intermediate and rate limiting step in fatty acid synthesis). The final product being, free palmitate that is capable of generating 129 moles of ATP when completely oxidized to produce energy during starvation.

### Shift from fatty acid synthesis to oxidation during feeding and fasting

As proposed by McGarry and Foster [6], the concentration of malonyl-CoA, the first committed intermediate in fatty acid biosynthesis, determines the rate of fatty acid oxidation. In the fed animal, where glucose is actively converted to fatty acids, the concentration of malonyl-CoA is elevated. Malonyl-CoA at micromolar concentrations inhibits hepatic carnitine palmitoyltransferase I (CPT I) thereby decreasing the transfer of fatty acyl residues from CoA to carnitine and their translocation into mitochondria. Consequently, β-oxidation is depressed.

When the animal changes from the fed to the fasted state, hepatic metabolism shifts from glucose breakdown to gluconeogenesis with a resultant decrease in fatty acid synthesis. The concentration of malonyI-CoA decreases, and the inhibition of CPT I is relieved. Furthermore, starvation causes an increase in the total CPT l activity and a decrease in the sensitivity of CPT I toward malonyl-CoA. Altogether, during starvation acylcarnitines are more rapidly formed and translocated into mitochondria thereby stimulating β-oxidation and ketogenesis.

### Fat body in invertebrates

Invertebrates, like other higher animals utilize glucose and fatty acids as sources of energy. Glucose, apart from its use in energy metabolism and heat generation during the cold, is important in the chitinous exoskeleton in insects while fatty acids are an important energy reserve for skeletal muscular activities such as hopping and flying, as well as secretion of pheromones and eicosanoids [7]. However, carbohydrate in insects is converted to trehalose (a disaccharide with O-α-D-Glucopyranosyl-(1 → 1)-α-D-glucopyranoside structure) and delivered to the fat body for storage in the form of glycogen [8]. Glycogen can be broken down to trehalose, which is secreted into the haemolymph for utilization [9]. Lipids on the other hand are digested to diacylglycerol. During the feeding period, most of the diacylglycerol is hydrolyzed to fatty acid and stored as triacylglycerol which can be converted back during molting, flight and oogenesis, and even stress conditions such as starvation, hypoxia and long-distance flight [8,10]. Amino acids from ingested proteins are also absorbed from the mid gut into the haemolymph from where they are transported to the fat body for storage.

The fat body in insects, being analogous to the adipose tissues and liver in the vertebrates is the main organ involved in energy metabolism and the major storage site for glycogen, lipid, and protein in insects. It is also the site of haematopoiesis and secretion of several immune components, antibacterial compounds and blood clotting proteins [11,12]. As the equivalent of mammalian adipose tissue, it shares similar developmental and functional pathways. It functions in sensing energy and nutrient availability, and coordinates the appropriate metabolic and survival responses. It is also the site of integration of pathogen responses with metabolic status [13]. DAG, TAG and cholesterol are transported by lipophorin from the gut to the fat body through the hemolymph and from the fat body to the various sites of utilization [14]. It also transports hydrocarbons such as n-pentacosane 3-methylpentacosane and 6, 9-heptacosadiene that are used in formation of cuticle from the site of their production in the oenocytes of the fat body [15]. The fat body also stores amino acids especially tyrosine in large quantity [16], and storage protein hexamerins as shown by the expression of the HEX 70a genes in the fat body, though the fat body is not the only site of storage of hexamerins because expression of HEX 70a genes that code for these storage proteins was found in other sites such as the gonads, suggesting that the protein has some reproductive importance [17].

Apart from the storage functions, the fat body in invertebrates is also involved in the regulation of hematopoiesis and innate immune homeostasis. The fat body in Drosophila has been shown to express the microRNA (miR-8) that is responsible for the production and regulation of AMPs (antimicrobial peptides) such as Drosomycin and Diptericin in those insects [18]. Many of these AMPs produced by the fat body appear to be targeted at specific types of microorganisms, for example, drosomycin and metchnikowins are active against fungi, defensins against Gram-positive bacteria, and attacins, cecropins, diptericins, and drosocins against Gram-negative bacteria [19]. The principal stimulus for the increased expression of AMP and lysozyme genes in insects is the identification of bacterial peptidoglycan in the cell wall of invading organisms.

The peptidoglycans are recognized by pattern recognition receptors (PRRs) known as peptidoglycan receptors proteins (PGRPs). The complex so formed stimulates the release of eicosanoids such as NFκB in the fat body in a manner similar to the sequence in mammals with consequent up-regulation of AMP genes and lysozymes followed by production of the antibacterial peptides against the invading organisms [19,20]. The fat body also maintains communication with hemocytes and other cells in the hemolymph via cytokines [19] for regulation of cellular immune response as well as nutrient sensing in order to prepare the insects against stress through the release of adequate nutrients [12].

An in depth understanding of the role of fat the body as the mediator of immune the system and pathogen sensing requires proper understanding of the roles of various PGRPs that have been identified. These include PGRPLE, PGRPLC and PGRPSA as well as the Toll and immune deficiency (Imd) pathways employed to produce the AMPs by the fat body in Drosophila immune response [21], after the recognition of invading pathogens ranging from fungi to gram positive and gram negative bacteria.

Insects do not have counterparts of mammalian B and T lymphocytes, and therefore the insect antimicrobial response does not exhibit a high degree of antigen targeting specificity. The inducible expression of antimicrobial peptides depends on the Toll and Imd signaling pathways. Fungal and Gram-positive bacterial infections in Drosophila stimulate primarily the Toll pathway, whereas Gram-negative bacterial infection stimulates primarily the Imd pathway [22]. The distinction in recognition of the bacteria and fungi is based on the recognition of specific peptidoglycans on the bacteria cell wall by PGRPs. Stimulation of the Toll pathway by Gram positive bacterial peptidoglycan is mediated through the short form of the extracellular peptidoglycan recognition protein (PRGP)-SA and PRGP-SD which act cooperatively in the presence of another pattern recognition protein - gram negative binding protein 1 (GNBP-1), which acts upstream simultaneously in the stimulation of the Toll pathway [22,23]. The stimulation of the Toll pathway by fungi does not depend on PGRP-SA, −SD or GNBP 1 but instead involves a serine protease, Persephone, and a protease inhibitor, Necrotic [22,24]. Both bacterial and fungal pathways converge to stimulate the protein Spätzle, a ligand of the Toll receptor. Activation of Toll leads to recruitment of three cytoplasmic proteins, which are (myeloid differentiation factor 88) MyD88, Tube and Pelle, to form the signaling complex underneath the cell membrane. The activated Pelle acts on the cytoplasmic Dorsal–Cactus and Dif (Dorsal related immune factor) –Cactus complexes. After signal-induced degradation of Cactus, Dorsal and Dif translocate to the nucleus and activate the expression of antimicrobial peptide genes. The AMP genes expressed include Drosomycin against fungal infections and diptericin, attacin against the Gram-positive bacteria.

Gram-negative bacteria on the other hand activate the Imd pathway. In peptidoglycan (PGN) of Gram-negative bacteria and Gram-positive bacilli, the lysine residue is replaced by meso-diaminopimelic acid (m-DAP). This second type of PGN (DAP-PGN) is sensed by PGRP-LC and PGRP-LE [25]. After recognition by the long class of PGRPs with long transcripts LC and LE they stimulate the Imd domain. Imd regulates TAK1 (Transforming growth factor-β-activated kinase 1), which then stimulates IKK-dependent cleavage and activation of Relish.

Imd also activates the FADD–Dredd pathway that also activates relish; this branch has a negative regulatory loop that involves the caspase inhibitor Dnr-1. Finally, there is the activation through TAK1 of the JNK pathway, which regulates early response genes, wound healing and melanization. The JNK pathway is also negatively regulated by relish. The Imd pathway is also generally inhibited by PGRP-LF via the JNK pathway [23] as well as caspar which blocks FADD-Dredd dependent cleavage of Relish, thereby preventing its translocation into the nucleus for stimulation of the antibacterial Diptericin genes as a form of feedback immune regulator in Drosophila [26]. By activating JNK, bacteria not only induce activation of antimicrobial peptide (AMP) genes, but also of genes encoding various cytokines and the cytoskeletal remodeling components required for phagocytosis [23].

In addition to activation of the Imd pathway, PGRP-LE also activates the Prophenoloxidase (proPO) cascade in Drosophila [21,27,28]. ProPO cascade is a proteolytic pathway responsible for formation of melanin around wounds to facilitate healing; and melanization or encapsulation [29] of invading pathogens. It involves the cleavage of prophenoloxidase to phenoloxidase by some serine proteinases systems that are activated by PGRP-LE. Phenoloxidase is thereafter converted to quinones and ultimately melanin in the presence of phenol and oxygen [30].

Chronic activation of the PGRPs in Drosophila, especially PGRP-LE portends serious danger for the organism due to the persistent production of the mediators of inflammatory response in the insects [21,31]. This is not far fetched because most of the intermediate products of proPO activation are either toxic to cells in large quantities or involved in ROS generation. Similar effects are also noticed in higher animals including man, because, most of the pathogen defense mechanism or systems exhibited in invertebrates are evolutionarily conserved [32]. For example, the Toll and Imd pathways for the synthesis of antimicrobial peptides in the Drosophila converge on relish, an NFκB homologue that is also found in vertebrates including man. Most of the other components of the Toll pathway including, Dorsal-related immunity factor (Dif) the Iκ B-like protein Cactus, via the recruitment of the myeloid differentiation factor 88 homologue (MyD88), the adaptor molecule Tube, and the IL-1R-associated kinase (IRAK)-like serine-threonine kinase - Pelle, are all members of NFκB homologues that are conserved in vertebrates including man. A new component of the Imd pathway, Akirin reported to be essential in the nuclear transcription of NFκB (Relish) dependent antimicrobial peptides in Drosophila is also conserved in the mice, where it is required in the downstream of TLR, TNF and IL-1β signaling, again at the level of the NF-κ B transcription factor for the production of IL-6 [33].

Abdelsadik and Roeder [31] reported that chronic activation of the salivary gland’s immune system via PGRP-LC and PGRP-LE activated Imd pathway resulted in reduced sizes in both, the salivary glands and of the entire drosophila in all its developmental stages. Just as persistent chronic inflammation results in growth retardation [34], reduction in life span, cachexia or chronic muscle wasting and metabolic syndrome in higher animals including man [35].

### The fat body in vertebrates

As enumerated by Gesta et al. [36], virtually all-animal species, from *C. elegans* to *Homo sapiens,* have found a way to store excess energy in the form of fat for future needs. *C. elegans* stores fat in intestinal epithelium and sharks store fat in the liver, both tissues of endodermal origin. But in most species, fat storage occurs in a mesodermal tissue, namely white adipose tissue (WAT). Location of these adipose tissues varies between species. For invertebrates, amphibians and many reptiles, the largest fat stores are intra-abdominal; in seals and whales, most fat is subcutaneous, whereas mammals and birds have both intra abdominal and subcutaneous white adipose tissue. The fat pad in cetacean whales is located in the head, ventro-lateral to the external auditory meatus and it is thought to be involved in sound wave transmission under water [37].

Early studies on energy storage in reptiles and amphibians involved the use of wet fat body weight as influenced by season and reproductive cycles, hibernation or estivation or analyses of the total triglyceride in dry fat body mass see [38] and [39]. It was concluded from these early studies that the major sites of fat storage in amphibian and reptiles are in the visceral fat body/abdominal fat pad, subcutaneous tissue and the tail. The animals’ dependence on or utilization of stored lipids varies from species to species. For example: stored lipid is utilized for both reproduction and winter maintenance in snakes whereas, it is used predominantly for reproduction in lizards [38].

In humans and lower mammals, fat is stored in the adipose tissue in various locations within the body such as intra abdominal depots around the omentum, intestines, and perirenal areas, as well as in subcutaneous depots in the buttocks, thighs, and abdomen. In addition, WAT can be found in many other areas, including in the retro-orbital space, face and the bone marrow [36]. Adipose tissue is made principally of white adipocytes, which are the primary site of triglyceride/energy storage, but also brown adipocytes, which are important in both basal and inducible energy expenditure in the form of thermogenesis. But brown adipocytes have not been reported in amphibians and reptiles [36]. Unlike in lower animals, which utilize stored fat for energy during starvation, poor food availability or during winter, storage of fat has been linked with reduced life expectancy [39] as a result of degenerative diseases and the development of metabolic syndrome(s) including conditions such as diabetes, hypertension, atherosclerosis and stroke [40]. White adipocytes have a spherical shape, and a single lipid droplet, surrounded by a reduced rim of cytoplasm, essentially occupying the fat cell volume. The nucleus and all the organelles are pushed to the periphery of the cell. White adipocytes are the only cells that may vary dramatically in size (from 10 to 180 μm diameter) under different patho/physiological conditions because of lipid accretion. Differences in fat cell size are depot-specific, for example in humans; the subcutaneous adipocytes are usually larger than adipocytes in visceral depots. Brown adipocytes on the other hand contain numerous cytoplasmic lipid droplets (unlike in the white adipocytes) with a central nucleus and a very large number of mitochondria characterized by typical laminar cristae generated by a prominent development of the inner mitochondrial membrane. Brown adipocytes are easily identified after labeling with antibodies for the uncoupling protein-1, a protein of the inner mitochondrial membrane responsible for the uncoupling of respiration and the thermogenic activity that characterizes these cells.

Enormous efforts have been dedicated to characterizing both white and brown adipose tissues in the mammalian body, including the precursors with the aim of elaborating the embryonic origin of the cells thereby of preventing or controlling obesity. Mesenchymal stem cells (MSCs) were identified as being capable of differentiating into adipocytes, osteoblasts, chondrocytes, myoblasts, and connective tissue. Although the exact number of intermediate stages between a mesodermal/ mesenchymal stem cell and a mature adipocyte is uncertain, it is believed that the MSC gives rise to a common early precursor (adipoblast), which in turn develops into committed white and brown preadipocytes that under appropriate stimulatory conditions differentiate into mature adipocytes of different types [36]. The transition from preadipocyte to adipocyte involves four stages: growth arrest, clonal expansion, early differentiation, and terminal differentiation. These stages are orchestrated by transcriptional cascades, which involve nuclear receptors - PPARγ (peroxisome proliferator-activated receptors) and members of the C/EBPs (enhancer binding proteins) family [41]. PPARγ plays an important role in adipogenesis and has been shown to be necessary and sufficient for adipocyte differentiation [42]. Two isoforms of PPARγ (PPARγ1 and PPARγ2) are generated by alternative splicing and promoter usage of the PPARγ gene. Although both are expressed in adipocytes, PPARγ2 has been regarded as a specific marker of fat. But PPARγ1 can compensate for loss of PPARγ2. It has also been observed that C/EBPα is equally required for white adipogenesis such that both C/EBPα and PPARγ control adipocyte differentiation, however, PPARγ appears to be dominant. PPARγ may also act as a tumor suppressor gene: it inhibits the growth of several cell types and induces apoptosis [43].

Summarily, the presence of PPARγ2 and other markers of terminal differentiation such as Glut4 and fatty-acid synthase serve as markers of mature adipocytes in WAT and BAT. Adipocytes also demonstrate insulin-regulated glucose uptake and metabolism [42]. In terms of specific localized marker that helps to differentiate WAT and BAT, it has been shown that WAT is characterized by the presence of leptin, whereas BAT is distinguished by the existence of UCP-1. In addition to adipocytes, fat pads contain preadipocytes, vascular cells, nerves, macrophages, and fibroblasts. Similar to white adipocytes, the differentiation of brown preadipocytes to brown adipocytes is controlled by a transcriptional cascade involving the transcription factors C/EBPs and PPARγ [36].

Besides serving as an energy storage depot, WAT has numerous functions. It has been accepted as the largest endocrine organ, secreting over 100 molecules described so far, they include a number of adipokines, such as leptin, adiponectin, tumour necrosis factor-alpha, interleukin (IL)-1b, IL-6, IL-8, IL-10, monocyte chemoattractant protein-1, macrophage migration inhibitory factor, nerve growth factor, vascular endothelial growth factor, plasminogen activator inhibitor-1 and haptoglobin [44]. The adipokines are highly diverse in terms of protein structure and of physiological function but many are linked to the immune system. They include classical cytokines e.g. tumour necrosis factor-alpha (TNFα), interleukin (IL6), growth factors e.g. transforming growth factor beta (TGFβ) and proteins of the alternative complement pathway (adipsin); they also include proteins involved in the regulation of blood pressure (angiotensinogen), vascular haemostasis e.g. plasminogen activator inhibitor-1 (PAI-1), lipid metabolism e.g. retinol binding protein, cholesteryl ester transfer protein (45), glucose homeostasis e.g. adiponectin, and angiogenesis e.g. vascular endothelial growth factor (VEGF). By far the most important secretory product of the adipose tissue is leptin which has not only been considered the principal modulator of hunger and satiety in the hypothalamus but also serves as a signal in reproduction and immunity [45]. Although WAT is the main source of the hormone, there has been a close correlation between body fat and the circulating leptin level but it is also secreted from other sites such as placenta, stomach, bone (osteoblasts), mammary epithelium and cells of the hair follicles [45] where it acts as a paracrine rather than as an endocrine hormone. Although adiponectin has been reported to possess anti-inflammatory action [46] most of the other adipokines are pro inflammatory cytokines that have been incriminated either directly or indirectly in the pathogenesis of metabolic syndrome predisposing to type 2 diabetes mellitus, atherosclerosis and cardiovascular disorders.

### The roles of the fat body in energy metabolism

#### Invertebrates

It has been stated and established from literature, earlier in this paper, that the insect fat body is analogous to the liver in vertebrates. It is responsible for storage of lipid, glycogen and protein. The fat body also senses, coordinates, and integrates multiple hormonal and nutritional signals involved in insect development and metamorphosis [8]. Apart from carrying out the crucial function of sensing energy and nutrient availability, and coordinating the appropriate metabolic and survival responses, it is also the site of coordination of responses to pathogen with metabolic status [12]. There is a high-energy demand in insects during several physiological processes such as larva-pupa formation, molting, vitellogenesis and other stressful conditions such as long distance migration, cold, hypoxia and starvation. Therefore, the insect must be able to store energy and make such available when the body demands for it. Mobilization of energy reserves is also involved in the acute stress response of insects. This response is controlled by the neurohormone octopamine, the invertebrate counterpart of noradrenaline [47].

Carbohydrate from the diet is digested in the insect mid gut and converted to trehalose, which is then exported into the hemolymph. Trehalose serves as a circulating sugar reservoir in the hemolymph, where trehalase hydrolyzes trehalose to glucose. Glucose is subsequently delivered to the fat body for storage and other tissues for utilization through various forms of glucose transporters [48]. In the fat body, glucose is converted to glycogen for storage. Glycogen can be mobilized and converted back to trehalose, which is secreted into hemolymph for utilization [9]. After mobilization from glycogen in the fat body, trehalose is utilized as energy source by short distance flyers such as cockroaches [49], it is also used as the initial source of energy by long distance flyers such as the migratory locust before they shift to lipid as energy source in prolonged flight [50].

The common pathway for synthesis of trehalose in insects involves combination of Glucose-6-phosphate (formed under the influence of phosphoglucomutase) and UTP (uridine triphosphate) to form trehalose-6-phosphate (T-6-P) and UDP under the influence of Trehalose synthase. T-6-P so formed is then dephosphorylated by Trehalose-6-phosphate phosphatase to form free trehalose and pyrophosphate [48,51]. The glucosyl donor may however vary from one organism to another, for example in *Streptomyces hygroscopicus*, and various other Streptomyces species that also produce trehalose-6-P, GDP-glucose, rather than UDP-glucose, serves as the glucosyl donor. See the review by Elbein et al. [51]. For utilization of trehalose as an energy source as mentioned earlier it must be reconverted into glucose in the presence of enzyme trehalase (α-glucoside-l-glucohydrolase), which hydrolyses trehalose to yield glucose. Glucose can then be used for glycogen or catabolized via glycolysis or the pentose phosphate pathway to produce ATP needed in flight muscle contraction.

Trehalose, like other disaccharides utilizes a transporter to cross cell membranes. The common transporter reported in the literature as being associated with trehalose is α-glucoside transporter AGT1, which promotes uptake of disaccharides, including trehalose, sucrose, and maltose, via an electrochemical proton gradient [52]. According to Kanamori et al. [53], insects also possess a facilitative transporter for trehalose TRET1, which regulates the level of trehalose in the haemolymph. A homologue of TRET1, N1st8, an H^+^ dependent trehalose transporter was characterized by Kikuda et al. [52] in the brown planthopper. It was observed to be responsible for reabsorption of trehalose in the Malphighian tubule.

From studies conducted on the migratory locust (*Locusta migratoria),* the release of trehalose from glycogen in the fat body is under the influence of adipokinetic hormone (AKH) released from the *Corpora cardiaca*, a neuroendocrine gland connected with the insect brain. AKH is released as need arises for increased energy substrate mobilization when stimulated directly by the brain through a specific nerve, known as *nervi corporis cardiaci I and II* (NCC I and II) that project directly from the protocerebrum of the insect into the glandular part of the *Corpora cardiaca* (a glandular neurohormone secretory organ caudal to the brain). AKH acts on the fat body via cAMP, calcium and inositol triphosphate mediated signal transduction. AKH signal transduction at the fat body target cells involves stimulation of cAMP production dependent on the presence of extracellular Ca^2+^. Additionally, the AKHs enhance the production of inositol phosphates including inositol (1,4,5) triphosphate, which may mediate the mobilization of Ca^2+^ from intracellular stores [54].

The major energy substrate, as mentioned earlier, utilized by insects and other invertebrates during starvation or high energy demanding feat such as flying is lipids in the form of diacylglyceride (DAG). Absorption of triglyceride after digestion of fat into the midgut in the form of chylomicrons has been studied extensively. Upon exposure to the haemolymph, DAG is ferried in the haemolymph into the fat body by an apolipoprotein known as high-density lipophorin (HDLp) an apolipoprotein produced by the insect fat body. Based on sequence homology between apolipophorin II/I in insects and apolipoproteins B in human, insect HDLp has been found to resemble mammalian LDL. Moreover, the size and the spherical shape of the HDLp particle are also similar to those of LDL. The resemblance of HDLp to LDL was recently extended by the identification of an insect LDL receptor (LDLR) family member that is capable of endocytic uptake of HDLp into fat body cells, indicating that receptor-mediated endocytosis constitutes an additional mechanism for lipid delivery.

Apolipoproteins are lipid-binding proteins in the blood, responsible for the transport of triacylglycerols, phospholipids, cholesterol, and cholesteryl esters between organs. Apolipoproteins (“apo” means “detached” or “separate,” designating the protein in its lipid-free form) combine with lipids to form several classes of lipoprotein particles, spherical aggregates with hydrophobic lipids at the core and hydrophilic protein side chains and lipid head groups at the surface. Various combinations of lipid and protein produce particles of different densities, ranging from chylomicrons and very low-density lipoproteins (VLDL) to high-density lipoproteins (HDL) may be formed from this combination.

Lipophorin is produced in the form of inactive precursor known as apolipophorin II/I. It acts as a lipid shuttle system, loading DAG in the gut in the presence of LTP (lipid transport protein), which acts catalytically to promote lipid export from the gut to lipophorin [55]. The DAG is then delivered to the fat body from the mid gut where it is converted to TAG for storage [56].

When stimulated by flight, insects release adipokinetic hormone from the corpora cardiac, which acts on the fat body to facilitate the release of TAG. AKH binds to an adenylate cyclase coupled receptor at the fat body inducing the conversion of ATP to cAMP. A putative hormone sensitive lipase is activated by cAMP and stored TAG is ultimately converted to sn-1, 2-diacylglycerol (DAG) the transport form of neutral glycerolipid which is loaded onto the HDLp, at the surface of the fat body in a process facilitated by lipid transfer protein (LTP). Concomitant with DAG uptake, an exchangeable apolipophorin, apoLp-III, associates with the particle. The resulting larger and less dense, low-density lipophorin (LDLp) particle, has a greater capacity to transport DAG from the fat body to the flight muscle [57].

In the flight muscle, DAG is hydrolyzed by lipophorin lipase present on the flight muscle to release free fatty acid that is used for oxidative phosphorylation. HDLp is regenerated after offloading the DAG and it is transported back to the fat body to continue the cycle.

Cholesterol is absorbed from the mid-gut lumen mainly in the free form and also transported by the high-density lipophorin (HDLp) exclusively in the free form [18]. HDLp, the sole carrier of cholesterol in haemolymph, distributes its sterol to different tissues, including the fat body where cholesterol is stored both in the free and the esterified forms. But the movement of cholesterol from haemolymph to tissues and the fat body is by passive diffusion or mass action [58].

Lipophorin has also been reported to be responsible for cellular lipid uptake in other tissues such as the brain and the oocytes. For instance, Parra-Peralbo and Culi [59] recently demonstrated that lipophorin receptor 1 and 2 (lpr1 and lpr2), two partially redundant genes belonging to the Low Density Lipoprotein Receptor (LDLR) family, are essential for the efficient uptake and accumulation of neutral lipids by oocytes and cells of the imaginal discs. Females lacking the lpr2 gene lay eggs with low lipid content and have reduced fertility, revealing a central role for lpr2 in mediating Drosophila vitellogenesis.

Insect apolipoprotein bears striking similarity with its human counterparts especially the 22 kDa N-terminal mammalian apolipoprotein B (apoB) and insect apoLp-II/I, which constitute the structural basis for the assembly of their respective lipoproteins, were shown to be homologues [60]. Additionally, microsomal triglyceride transfer protein (MTP), a dedicated cofactor through which both apolipoproteins acquire lipids, is another member of the same family as apo B and apoLp-II/ I. Based on the sequence homology between apoLp-II/I and apoB, insect HDLp resembles mammalian LDL [61]. Insect apolipoprotein precursors belong to the large lipid transfer protein (LLTP) superfamily that emerged from an ancestral molecule and includes mammalian apoB, MTP, and vitellogenin [62]. The LLTP domain shared by these proteins comprises a large N-terminal domain of about 1,000 amino acids containing a large lipid pocket that is proposed to act as a lipid store and to transfer lipids to the apolipoprotein in a coordinated manner [63].

Maintaining a balance between energy storage and utilization as well as a shift in focus of where the energy is channeled requires that the organism have a switch between conservation and utilization. This role in the fat body is performed by MEF2 (myocytes enhancer factor-2), a transcription factor found in muscles, neuron and immune cells [64]. MEF2 binds directly to the promoters or enhancers of the majority of muscle specific genes and interacts with members of the MyoD family of basic helix–loop–helix (bHLH) proteins to activate the skeletal muscle differentiation program during embryogenesis. It also supports neuronal and immune cell differentiation as well as protect the cells against apoptotic cell death [64]. Furthermore, MEF2 transcription factor plays central roles in the transmission of extracellular signals to the genome and in the activation of the genetic programs that control cell differentiation, proliferation, morphogenesis, survival and apoptosis of a wide range of cell types [65]. Serving as endpoints for multiple signaling pathways and thereby confer signal-responsiveness to downstream target genes e.g. MAP kinase signaling pathways in various animal species including man.

In a recent study by Clark et al. [66] MEF2 was observed to act as an immune-metabolic switch in the fat body. MEF2 is phosphorylated at a conserved site in healthy flies and promotes expression of lipogenic and glycogenic enzymes. Upon infection, this phosphorylation is lost, and the activity of MEF2 changes. MEF2 now associates with the TATA binding protein to bind a distinct TATA box sequence and promote antimicrobial peptide expression. The choice between immune and metabolic target genes is dictated by phosphorylation of MEF2 at a conserved site. In healthy animals, MEF2 is phosphorylated at T20 and promotes expression of its metabolic targets, whereas infection results in T20 dephosphorylation and association with the TATA-binding protein (TBP) at a distinct TATA sequence found in the core promoters of antimicrobial peptides. The absence of T20-phosphorylated MEF2 promotes the loss of anabolic transcripts in flies with Gram-negative bacterial infection [66].

The T20 region is conserved in *Drosophila, C. elegans*, and all mouse and human MEF2 homologues, and it matches a crude consensus site for the insulin effector kinase (AKT) and p70 S6 kinase [66]. S6K in vivo activates MEF2-T20 kinase and may be responsible for promoting expression of the enzymes of lipogenesis and glycogenesis in healthy, well-fed animals. [67], i.e. S6K enhances anabolism and repress catabolism in response to nutrient signals [68]. It must be noted that TAK1 is required for formation of the MEF2-TBP complex upon Gram-negative bacterial infection [69] and subsequent production of AMPs via the Imd pathway. Clark et al. [66] also suggested that wasting seen after infection may be due, in part, to the requirement for MEF2 to serve different transcriptional functions in different conditions; the MEF2 immune-metabolic transcriptional switch may be a mechanistic constraint that forces the fly into metabolic pathophysiology in contexts of persistent immune activation. Other pathways may also be involved in wasting associated with Gram-negative bacterial infections. For example, Dionne et al. [70] reported that reduction in the insulin effector kinase – Akt was responsible for excessive activity of Gsk 3 and FOXO and subsequent depletion of energy store in Drosophila infected with *Mycobacterium mirium.*

### Vertebrates

Lipid levels in an adipocyte are the net result of several different processes, including lipoprotein hydrolysis, fatty acid uptake/de novo fatty acid synthesis, and fatty acid esterification. Lipid mobilization and utilization are two processes intimately intertwined. Lipid mobilization from adipose tissue depots results in the release of FFAs into the blood where they can be transported and taken up by tissues for energy. Lipid utilization requires the uptake of FFAs into the cell where they can be oxidized via the mitochondria through the process of β-oxidation [71]. Morphology and site of deposition of fat in major vertebrates has been well elucidated by Pond [72]. Subcutaneous fat is poorly developed in reptiles and absent from amphibians presumably because in the latter, concentrations of fat cells under the skin would be incompatible with the role of the skin as a respiratory organ. Paired abdominal fat bodies are well developed in many amphibians and reptiles, except chelonians [72].

Triacylglycerol (TAG) is the most concentrated form of energy available to biological tissues [73]. The adipocytes are an important example of a reservoir of lipids. The TAG stored within intracellular lipid droplets in adipocytes can be rapidly mobilized by hormone-sensitive lipase in vertebrates [74] into the blood, which transports it to different tissues. McCue [75] also demonstrated in squamate reptiles that stored lipids especially triglyceride is the major energy source during starvation using FAME (fatty acid methyl esters) analyses conducted on the total body lipids of rattlesnakes (*Crotalus atrox*), ratsnakes (*Elaphe obsoleta*), pythons (*Python regius*), boas (*Boa constrictor*), true vipers (*Bitis gabonica*), and monitor lizards (*Varanus exanthematicus*).

### Possible nexus between fat body metabolism and diseases

#### The nexus between obesity, inflammation and insulin resistance

As observed by Hoptamisligil [12], among the most critical processes to species survival are the ability to withstand starvation and the capacity to mount an effective immune response to pathogens. The former selects for energy efficiency and favours the storage of excess calories when access to food is intermittent. However, in the presence of a continuous nutritional surplus, this once advantageous metabolic state could set the stage for excess adiposity and its associated problems. One of these problems associated with excess adiposity is obesity. In essence, energy imbalance in either extreme has its complications: starvation being associated with immunosuppression because the body response against pathogen requires metabolic support and energy redistribution [76] while obesity is associated with inflammatory diseases [77]. For instance, lipolysis in adipose tissue during an inflammatory response is primarily driven by hormone-sensitive lipase (HSL), which is regulated either by alteration in its phosphorylation state or by induction of gene expression. Several cytokines that induce lipolysis, including TNF, IFN-α, and IFN-γ produce a marked decrease in HSL mRNA.

Obesity is a form of chronic metabolic overload [77] and has been associated with production of leptin, adiponectin and other pro- and anti-inflammatory cytokines. Though there are several pathways through which inflammation occurs, the irony of obesity-induced inflammation is that of an overwhelmed pet or servant that is torn between two masters. This is due to the fact that adipose tissue produces adiponectin, which has anti-inflammatory action and also promotes insulin sensitivity. But its rate of secretion is inversely proportional to the degree of obesity/quantity of fat. Adipose tissues also produce IL 1Receptor α (IL-1Rα), which also shows anti-inflammatory activities. But the number and quantity of pro-inflammatory cytokines including TNFα, IL −6, −1, −8, −10 and −18, vifatin and resistin, MIF (macrophage migration inhibition factor), MCP 1 (monocytes chemo-attractant protein 1), M-CSF, TGFβ, C reactive protein and haptoglobin produced by adipose tissues far overwhelms any anti-inflammatory activities of the former.

Apart from the production of adipokines, elevated levels of apoB-containing lipoproteins are a hallmark of metabolic syndrome, a pathological condition comprising wide-ranging dysfunctions in different tissues. These include obesity, diabetes, heart disease and increased risk of dementia [55].

### The role of TLR in inflammation and insulin resistance

Toll-like receptors (TLRs) function as pattern recognition receptors in mammals; they play essential roles in the recognition of microbial components. TLRs may also recognize endogenous ligands induced during inflammatory response. TLRs are type I integral membrane glycoproteins, and on the basis of considerable homology in the cytoplasmic region, they are members of a larger superfamily that includes the interleukin-1 receptors (IL-1Rs). The mechanism involved in TLRs induced inflammatory signaling and the release of inflammatory cytokines by NFκB has been extensively described. Akira and Takeda [32] postulated that stimulation of TLRs by lipopolysaccharides, peptidoglycan flagelin, heat shock proteins, double and single stranded RNA, or even synthetic compounds such as imidazoquinoline etc. triggers the association of MyD88 (myeloid differentiation primary-response protein 88), which in turn recruits IRAK4 (IL-1R-associated kinase 4), thereby allowing the association of IRAK1. IRAK4 then induces the phosphorylation of IRAK1. TRAF6 (Tumour necrosis factor receptor associated factor 6) is also recruited to the receptor complex, by associating with phosphorylated IRAK1. Phosphorylated IRAK1 and TRAF6 then dissociate from the receptor and form a complex with TAK1 (transforming growth factor β activated kinase 1). TAK1 ultimately phosphorylates both mitogen-activated protein (MAP) kinases and the IKK complex (inhibitor of nuclear factor κB (IκB) kinase complex), which consists of IKK α, IKK β and IKK γ. The IKK complex then phosphorylates IκB. This allows NF-κB to translocate to the nucleus and induce the expression of its target genes.

Lipids play an important role in direct activation of the innate immune system via TLR signaling. Free fatty acids are important ligands for TLR-2 and TLR-4. For example, Davies et al. [78] reported that TLR 4 is activated by saturated fatty acids such that TLR4 deficient mice showed markedly lower circulating concentrations of MCP1 and much less NFκB protein in nuclear extracts prepared from adipose tissue. TLRs, therefore, represent a direct molecular link between hyperlipidemia (a central clinical feature of obesity) and activation of the innate immune system [78]. TNFα levels are increased in adipose tissue and serum in murine and human obesity. In addition to its role in inflammation, TNFα induces insulin resistance in a wide range of cells including adipocytes, through multiple mechanisms, including down regulation of expression and phosphorylation of insulin receptor, IRS-1 and IRS-2, and inhibition of GLUT 4 expression and translocation [79]. Increased expression of inflammatory mediators has also been observed in visceral fat of obese humans. These cytokines have been shown to disrupt insulin signaling through several mechanisms, including induction of the suppressors of cytokine signaling (SOCs) family of proteins, which have been reported to inhibit insulin receptor kinase activity, interfere with binding of insulin receptor substrate IRS1 and IRS2 to the insulin receptor, and promote IRS degradation [80]. Furthermore, cytokines could also activate inflammatory signaling via c-Jun N-terminal kinase (JNK) and inhibitor of kappa B kinase (IKK B) pathways in both immune and neighbouring non-immune cells [80].

Inflammation as a result of trauma, ischaemia–reperfusion injury or chemically induced injury typically occurs in the absence of any microorganisms and has therefore been termed ‘sterile inflammation’ [81]. Similar to microbial induced inflammation, sterile inflammation is marked by the recruitment of neutrophils and macrophages and the production of pro-inflammatory cytokines and chemokines, notably TNFα and IL1. Examples include chronic inhalation of sterile irritants, such as asbestos and silica, can lead to persistent activation of alveolar macrophages and result in pulmonary interstitial fibrosis. In ischaemia–reperfusion injury, as seen with myocardial infarction and stroke, gout and pseudogout, in which the deposition of monosodium urate and calcium pyrophosphate dihydrate crystals in the joints results in acute neutrophilic infiltration followed by chronic inflammation, fibrosis and cartilage destruction. In Alzheimer’s disease, neurotoxicity is associated with activated microglial cells adjacent to β amyloid containing plaques that generate ROS in addition to pro inflammatory cytokines.

PRRs also recognize non-infectious material that can cause tissue damage and endogenous molecules that are released during cellular injury [32]. These endogenous molecules have been termed damage associated molecular patterns-DAMPs [81]. DAMPs are released from necrotic or damaged tissues or in condition of cell stress and injuries; they include heat shock protein, purine metabolites such as ATP and uric acid, hyaluran, heparin, biglucan and β amyloid. Some inflammatory cytokines such as IL-1α and IL-33 may also act as DAMPs. Exogenous DAMPs include asbestos and silica [81].

DAMPs after recognition stimulate inflammatory responses via TLRs, because TLRs are important in regulation of innate immunity against both infectious and sterile inflammatory stimuli. Furthermore, since free fatty acids are important ligands for both TLR 2 and TLR 4, this indicates that TLRs are important links between hyperlipidemia (a hallmark of obesity) and activation (perpetual) of innate immune system as TLRs up regulate and activate the NFκB system [79].

DAMPs can also be recognized by receptors of advanced glycation end products (RAGE), a trans membrane receptor that is expressed by immune cells, endothelial cells, cardiomyocytes and neurons [82]. It detects advanced glycation end products (AGEs) that arise from non-enzymatic glycation and oxidation of proteins and lipids. These products can accumulate under conditions of high oxidant stress and are found at elevated levels in chronic inflammatory disease states. Activation of RAGE by its ligands results in the upregulation of several inflammatory signaling pathways, including, NFκB, signaling pathways, which lead to induction of pro-inflammatory cytokines such as TNF [81].

### The nexus between adipocytes and macrophages in inflammatory cytokine secretion

Like macrophages, the adipocyte is exquisitely sensitive to infectious disease agents and cytokine-mediated inflammatory signals; it expresses a host of receptors, enabling it to sense the presence of pathogens and inflammation, and on stimulation of these receptors, it activates multiple inflammatory signal transduction cascades, and induces and secretes a number of potent inflammatory cytokines and acute phase reactants. Adipocytes are sensitive to the effects of tumor necrosis factor-α (TNF-α), which, through its p55 and p75 TNF receptors, stimulates NF-κB, extracellular signal-regulated kinase, and p38 mitogen-activated protein kinases PI-3 kinase and jun-N-terminal kinase (JNK) cascades [83]. Obesity also overloads the functional capacity of the endoplasmic reticulum (ER) leading to ER stress which has been reported to activate inflammatory signaling that contribute to insulin resistance. Hyperglycemia also increases mitochondrial reactive oxygen species (ROS) generation, which is usually elevated in obesity. ROS also enhances inflammatory signaling. The central mechanism of this stress induced metabolic syndrome lies in the ability of these stressor factors and cytokines to activate JNK, which phosphorylates IRS-1 (insulin receptor substrate 1) on Ser 307, thus impairing insulin action [77]. In obesity, JNK activity has been reported to be elevated in the liver, muscle and adipose tissue. Loss of JNK1 receptors has been found to prevent development of insulin resistance in mouse [84].

Most of the inflammatory cytokines produced by adipose tissues are also produced by macrophages. Since macrophages infiltrate adipose tissues, they may therefore be the source of the adipokines. The infiltration of adipose tissue especially around necrotic cells was initially thought to be attracted by secretion of chemo attractants by adipocytes and preadipocytes [77] and meant for clearance of necrotic debris alone [85], it has however been observed by Charriere et al. [86] that since macrophages accumulate lipids like the adipocytes to become foam cells, they may, therefore, have similar ancestors [87]. In actual fact, pre-adipocytes have been reported to show phagocytic and other antimicrobial properties and may even differentiate to macrophages in tissue. We can therefore conclude that production of inflammatory cytokines by adipose tissue may not be the responsibility of the adipocytes alone, but in conjunction with macrophages.

The infiltrating macrophages in obesity have also been incriminated in development of cancer cachexia as it causes an increase in the release of other inflammatory cytokines such as IL-6 and IL-1b in addition to TNF-α and prostaglandin E_2_[35,88]. Macrophage-derived TNF-α enhances lipolysis and down regulates peroxisome proliferator-activated receptor γ (PPARγ)- mediated triglyceride (TG) biosynthesis and storage in adipocytes [89]. Although, the adipocytes attempt to inhibit the lipolytic action of TNF-α by producing IL-10 and IL-1ra, which act against MCP-1 (monocyte chemo-attractant protein 1) to prevent formation of TNF-α, IL-6, and IL-1b [90], the action may not always yield desired result.

### The missing link in evaluation of the fat body in vertebrates

Adipose tissue has been extensively studied, not just as a storage organ for lipids but as an energy sensor and in immune and inflammatory response mediation. The damaging effects of obesity and avenue for correction of both obesity and the associated metabolic anomalies are still being investigated. It might therefore be necessary for efforts to be focused on the insect fat body, especially on how it is able to store lipids without the lipid showing any damaging effect during storage and lipolysis for energy substrate mobilization in insects.

Recently, lipid droplets which are the intracellular energy storage organelles have been found to be involved in energy homeostasis as passive fat storage [91], membrane and lipid hormone precursors in lipometabolism [92] and as sites of fatty acid scavenging to protect cells from lipoapoptosis [91]. In a recent study by Anand et al. [93], lipid droplets bound to histone have been shown to possess antibacterial activities and capable of protecting the cell from bacterial invasion and multiplication [94]. Histones (chromatin protein) are found in large numbers in most animal cells, they assist in the folding of DNA strands into compact and robust structures inside the nucleus. Free histones in the cytosol however can be extremely damaging to cells because unconfined histones have been reported to cause genomic instability, hypersensitivity to DNA-damaging agents, and lethality [95]. Cells therefore minimize excess free histones in the cytosol by active mechanisms. So, most species have developed mechanisms to detect and degrade free histones in the cytosol [93]. However, histones were observed by these authors to be sequestered to lipid droplets, and were released in response to the presence of LPS from the bacterial cell wall. These histones kill Gram-positive as well as Gram-negative bacteria, both in vitro and in vivo [93]. The sites of these controls on energy homeostasis have been suggested to be the associated proteins of these lipid droplets known as lipid droplet proteomes, one of which is the PAT (Perilipin, ADRP and TIP47) domain proteome family. This proteome is made up of Perilipin, which modulates the rate of lipolysis, ADRP (adenine derived differentiation related protein) that has been reported to be involved in intracellular lipid transport and TIP47 (47-kDa tail-interacting protein). The major importance of the PAT protein domain is that, it has been reported to be evolutionarily conserved, even from invertebrate to humans. For instance, a perilipin homologue, LSD-1 (lipid droplet protein 1) or perilipin 1 [96] has been isolated in Drosophila and found to be responsible for regulation of energy storage in the fat body and control of lipid droplet transportation [97]. It has also been found to regulate sleep homeostasis in Drosophila [98]. Perilipin is a critical regulator of lipolysis in vertebrate adipocytes and, depending on its phosphorylation level, it can prevent or stimulate triglyceride hydrolysis. The two key enzymes involved in TAG mobilization within adipose tissue are lipoprotein lipase (LPL) and hormone-sensitive lipase (HSL). HSL actively releases fatty acids from TAG stored within adipocyte cytosolic lipid droplets and responds to the hormonal balance of insulin and catecholamines which transmit information about the body’s physiological and nutritional status. Perilipin is thought to be crucial in the regulation of this process. cAMP-mediated phosphorylation of perilipin (which normally covers a large proportion of the surface area of the lipid droplet to protect it from lipolytic attack by HSL), results in its redistribution, thereby allowing access of the activated HSL to the droplet TAG substrate [99]. Perilipin thus modulates the rate of adipocyte lipolysis by acting both as a barrier and attachment site for lipases in a phosphorylation-dependent manner.

Insect genomes encode two PAT proteins, Lsd1 and Lsd2. Like perilipin in vertebrates, LSD1 is the major phosphoprotein of lipid droplets after hormone stimulation of lipolysis [100]. In actual fact, phosphorylation of LSD1 is responsible for most of the lipolytic response elicited by adipokinetic hormone (AKH) in *M. sexta*[7]. Lsd2 seems to be involved in promoting lipid accumulation, whereas Lsd1 is involved in activation of lipolysis. Lsd2 is expressed during all developmental stages and is required for normal storage of triglyceride in the fly. PAT domain protein LSD-2 of Drosophila not only serves a Perilipin-like function in adult fat body cells but also operates in the control of embryonic lipid droplet transport along the cytoskeleton [91]. Overexpression and deletion of Lsd2 in Drosophila leads to an increase and a reduction of the triglyceride content, respectively. The lack of Lsd2 is also associated with a decrease in the lipid content of the embryo, suggesting that it is important for the transfer of lipids to the developing oocyte [7].

Overexpression of ADRP has also been associated with expansion of lipid droplet pools and increased cellular triacylglycerol (TAG) mass. For examples, over-expression of an ADRP fusion protein in Swiss-3 T3 cells resulted in increased triacylglycerol (TAG) content, even when the cells were cultured in de-lipidated serum [100].

According to Beller et al. [91], defects or mutation in some of the genes that code for these proteomes especially in SMP30 knock-out mutant mice and adp60 mutant flies have been shown to lead to lipid accumulation/obesity. In fact lipid accumulation in these insects as suggested from their findings in wild type *Drosophila melanogaster* Genotype-specific differences in the lipid droplet sub-proteome composition might therefore be more pronounced during the adult stage. Thus, identifying genetic predisposition to obesity may lie in identifying the fat droplet proteomes and sub-proteomes and their defects, early in life as guide for counseling people who are predisposed to obesity. In support of this observation, its been reported that insects with mutation in lipid droplet protein 1 (LSD1) also known as perilipin 1 have adult onset obesity [96].

### Concluding remark

The role of the fat body in invertebrates as a storage organ and immune mediator has been observed to be comparable to that of the adipose tissue in the vertebrates, the solution to obesity and obesity related complications therefore, may lie in further investigation of the role of the fat droplet proteomes in the control of lipolysis and lipid accumulation in the Drosophila especially the PAT proteins LSD 1 (Perilipin) LSD 2, and ADRP.

## Abbreviations

CPT I: Carnitine palmitoyltransferase I; AKH: Adipokinetic hormone; DAG: Diacylglycerol; Hex: Hexamerins; AMP: Antimicrobial petides; PRR: Pattern recognition receptors; PGRPs: Peptidoglycan receptor proteins; TNFκB: Nuclear factor kappa B; PAI 1: Plasminogen activation inhibitor factor 1; WAT: White adipose tissue; MSC: Mesenchymal stem cell; PPRAγ: Peroxisome proliferator-activated receptor gamma; C/EBPs: Enhancer binding proteins; BAT: Brown adipose tissue; UCP1: Uncoupling protein 1; IL: Interleukins; TGFβ: Transforming growth factor beta; T-6-P: Trehalose 6 phosphatase; UDP: Uridine diphosphate; UTP: Uridine triphosphate; AGT1: α glucoside transporter 1; TRET1: Trehalose transporter 1; HDLp: High density lipophorin; LDL: Low density lipoprotein; VLDL: Very low density lipoprotein; VHDL: Very high density lipoprotein; LTP: Lipid transport protein; TAG: Triacylglycerol; MTP: Microsomal triglycerol transfer protein; LLTP: Large lipid transfer protein; FFAs: Free fatty acids; FAME: Fatty acid methyl esters; HSL: Hormone sensitive lipase; IFN: Interferon; MCP1: Monocyte chemo-attractant protein; MIF: Macrophage migration inhibition factor; M-CSF: Monocyte colony stimulating factor; TGFβ: Tissue growth factor beta; JNK: c-jun-N-terminal kinase; IRS1: Insulin receptor substance 1; PAT: Perilipin-ADRP-TIP47 domain; ADRP: Adenine derived differentiation related protein; TIP47: 47 kDa tail interacting protein; LSD1: Lipid droplet protein 1; FAAD: Fas associated protein with death domain; TAK1: Transforming growth factor β activated kinase; GNBP1: Gram negative binding protein 1; MyD88: Myeloid differentiation factor 88; TRAF6: Tumour necrotic factor receptor associated factor 6; MAP: Mitogen activated protein kinase; IKK: Inhibitor of nuclear κB; FOXO: Forkhead box O transcription factor; AKT: Insulin effector kinase; Gsk 3: Glycogen synthase kinase 3; TATAAT: TATA- A + T rich sequence; TBP: TATA binding protein.

## Competing interests

The authors declare that there is no conflict of interest whatsoever in the publication of this paper.

## Authors’ contributions

JP conceived the idea, OI got the materials and wrote the manuscript while JP and RA read through and corrected the manuscript. All authors read and approved the final manuscript.
